# Neoadjuvant versus adjuvant treatment: which one is better for resectable esophageal squamous cell carcinoma?

**DOI:** 10.1186/1477-7819-10-173

**Published:** 2012-08-25

**Authors:** Yaping Xu, Xinmin Yu, Qixun Chen, Weimin Mao

**Affiliations:** 1 Department of Radiation Oncology, Zhejiang Cancer Hospital, Hangzhou, 310022, Zhejiang, People’s Republic of China; 2 Department of Medical Oncology, Zhejiang Cancer Hospital, Hangzhou, 310022, Zhejiang, People’s Republic of China; 3 Department of Thoracic Surgery, Zhejiang Cancer Hospital, Hangzhou, 310022, Zhejiang, People’s Republic of China

**Keywords:** Esophageal cancer, Squamous cell carcinoma, Neoadjuvant therapy, Adjuvant therapy, Chemotherapy, Radiotherapy, Chemoradiotherapy

## Abstract

Esophageal cancer is the eighth most common cancer worldwide, and especially in some areas of China is the fourth most common cause of death and is of squamous cell carcinoma (SCC) histology in >90% of cases. Surgery alone was the mainstay of therapeutic intervention in the past, but high rates of local and systemic failure have prompted investigation into multidisciplinary management. In this review, we discuss the key issues raised by the recent availability of esophageal SCC treatment with the addition of chemotherapy, radiotherapy, and chemoradiotherapy to the surgical management of resectable disease and discuss how clinical trials and meta-analysis inform current clinical practice. None of the randomized trials that compared neoadjuvant radiotherapy or chemotherapy with surgery alone in esophageal SCC has demonstrated an increase in overall survival in those patients treated with neoadjuvant radiotherapy or chemotherapy. Neoadjuvant chemoradiotherapy has been accepted recently for esophageal cancer because such a regimen offers great opportunity for margin negative resection, improved loco-regional control and increased survival. The majority of the available evidence currently reveals that only selected locally advanced esophageal SCC are more likely to benefit from the adjuvant therapy. The focus of future trials should be on identification of the optimum regimen and should aim to minimize treatment toxicities and effect on quality of life, as well as attempt to identify and select those patients most likely to benefit from specific treatment options.

## Review

### Introduction

Esophageal cancer is the most rapidly increasing tumor type in the world [1,2]. Globally, esophageal cancer is the eighth most common malignancy and sixth most fatal disease, with approximately 460,000 new diagnoses and > 380,000 deaths annually [3]. More than one-half of new cases in western countries have adenocarcinoma (AC) of the lower esophagus or gastroesophageal junction; and > 90% patients in eastern Asian countries have squamous cell carcinoma (SCC) [3,4].

Despite improvements in surgical and radiotherapy (RT) techniques and refinements of chemotherapeutic regimens, long-term survival, even from localized esophageal cancer, remains poor. Surgery alone was the mainstay of therapeutic intervention in the past, but high rates of local and systemic failure have prompted investigation into the multidisciplinary management. Treatment paradigms differ between western and Asian countries, but the unifying theme that has emerged in the past decade implies that surgery alone can no longer be considered the standard of care. In attempts to improve the survival rate, the multidisciplinary management involved various combinations and sequences of all modalities including surgery, RT, chemotherapy (CT), and chemoradiotherapy (CRT).

The prognosis was different slightly between histological subtypes. Prior studies have shown that, in comparison with patients with AC, those with esophageal SCC have worse prognosis [5-7]. This difference is probably due to a different pattern of lymphatic spread and a greater tendency for the disease to spread locally [8,9], but more importantly due to the location of the primary tumor. SCC is usually a proximal lesion, with 75% of these cancers found to have contact with the tracheobronchial tree, while 94% of AC are below the tracheal bifurcation [10].

Neoadjuvant CRT is currently the standard of care for esophageal AC in many western countries. However, the optimal treatment strategy for resectable esophageal SSC is still a controversial topic. In this review, we discuss the key issues raised by the recent availability for esophageal SCC treatment with the addition of RT, CT, and CRT to the surgical management of resectable disease, and discuss how clinical trials and meta-analysis inform current clinical practice.

### Neoadjuvant treatments

#### Neoadjuvant radiotherapy

There have been five phase III randomized trials comparing neoadjuvant RT with surgery alone [11-15] in esophageal SCC (Table 1) and none has demonstrated an increase in resectability or overall survival (OS) in those treated with neoadjuvant RT. Although Nygaard and colleagues reported a 3-year OS benefit, this was only after pooling patients who had received neoadjuvant RT with those who had also received neoadjuvant CRT, as there was no significant difference in survival found otherwise [14]. Meta-analysis of the five above mentioned randomized trials only proved a nonstatistically significant trend (hazard ratio (HR): 0.91, 95% CI: 0.80 to 1.04) in favor of neoadjuvant RT [16].

**Table 1:** Randomized controlled trials of neoadjuvant radiotherapy versus surgery alone for esophageal squamous cell carcinoma

**Study**	**Histology**	**SCC (%)**	**Treatment**	***n***	**MS (months)**	**5-year OS (%)**	***P *value**
Launois and colleagues [11]	SCC	100	NART 40 Gy	77	10	10	NS
			Surgery	57	12	12	
Gignoux and colleagues [12]	SCC	100	NART 33 Gy	106	11	11	NS
			Surgery	102	11	10	
Arnott and colleagues [13]	AC/SCC	36	NART 20 Gy	90	8	9	NS
			Surgery	86	8	17	
Nygaard and colleagues [14]	SCC	100	NART 35 Gy	48		21^a^	NS
			Surgery	41		9^a^	
Wang and colleagues [15]	SCC	100	NART 40 Gy	104		35	NS
			Surgery	102		30	

^a^Three-year overall survival (OS). *AC* adenocarcinoma, *MS* median survival, *NART* neoadjuvant radiotherapy, *NS* not significant, *SCC* squamous cell carcinoma.

A recent meta-analysis of trials including data from 1,033 patients, 33% of whom presented SCC, revealed significantly improved median OS (27 months vs. 18 months, *P* < 0.0001) and cause-specific survival (35 months vs. 21 months, *P* < 0.0001) with neoadjuvant RT. These results support the use of neoadjuvant RT for patients with esophageal cancer, but patients with SCC were not analyzed separately [17]. Given the lack of demonstrated significant benefits of neoadjuvant RT in SCC patients, a preoperative neoadjuvant radiation treatment strategy is currently not recommended in the SCC population.

#### Neoadjuvant chemotherapy

The theoretical advantages of adding CT to the treatment of esophageal cancer are potential tumor down-staging prior to surgery, as well as targeting micrometastatic disease and thus decreasing the risk of distant spread. Potential drawbacks include morbidity and mortality associated with CT toxicity, selection of drug-resistant clones, and delay in definitive surgical management.

Early randomized trials using cisplatin, fluorouracil (5-FU), bleomycin, vindesine and their combinations were conducted in patients with SCC of the esophagus [14,18-21] (Table 2). However, these trials were underpowered to detect any difference in outcome with the addition of CT to surgery. Three-year OS rates ranged from 3 to 43%, despite the use of very similar CT regimens. Two large randomized studies that evaluated neoadjuvant CT with cisplatin and 5-FU for operable esophageal AC or SCC had contrasting results. The smaller Radiation Therapy Oncology Group trial 8911 (US Inter group 113) study reported no survival benefit, with a median survival of 14.9 months in patients treated with CT compared with 16.1 months in those receiving surgery alone (*P* = 0.53) [22]. Only 31% of patients in the larger multi-institutional Medical Research Council OE02 study had SCC, reflecting the increasing relative incidence of esophageal AC in the UK. Five-year OS was increased by 6% from 17.1 to 23% (HR: 0.84, 95% CI: 0.72 to 0.98; *P* = 0.03) with the addition of CT to surgery [23]. The discrepancy between the results of the Radiation Therapy Oncology Group trial 8911 [22] and OE02 [23] studies is due to the larger proportion of patients with SCC in the former trial who do not appear to significantly benefit from this strategy.

**Table 2:** Randomized controlled trials of neoadjuvant chemotherapy versus surgery alone for esophageal squamous cell carcinoma

**Study**	**Histology**	**SCC (%)**	**Treatment**	***n***	**MS (months)**	**3-year OS (%)**	***P *value**
Schlag [18]	SCC	100	CF	22	7		NS
			Surgery	24	6		
Nygaard and colleagues [14]	SCC	100	BC	44	7	3	NS
			Surgery	41	7	9	
Maipang and colleagues [19]	SCC	100	BVC	24	17	31	NS
			Surgery	22	17	36	
Law and colleagues [20]	SCC	100	CF	74	17	40	NS
			Surgery	73	13	13	
Ancona and colleagues [21]	SCC	100	CF	47	25	34^a^	NS
			Surgery	47	24	22^a^	
Kelsen and colleagues [22]	AC/SCC	54	CF	213	15	19^a^	NS
			Surgery	227	16	20^a^	
Allum and colleagues [23]	AC/SCC	31	CF	400	17	43	<0.01
			Surgery	402	13	34	

^a^Five-year overall survival (OS). *AC* adenocarcinoma, *BC* bleomycin + cisplatin, *BVC* bleomycin + vindesine + cisplatin, *CF* cisplatin + fluorouracil, *MS* median survival, *NS* not significant, *SCC* squamous cell carcinoma.

In 2008 a Japan Clinical Oncology Group study (JCOG 9907) comparing adjuvant and neoadjuvant CT was reported, and those results showed that neoadjuvant CT induced down-staging and R0 reduction and improved OS without additional serious adverse events [24]. However, the efficacy of neoadjuvant CT was not compared with surgery alone in this randomized trial. Prior to the JCOG 9907 study, the authors had compared adjuvant CT and surgery alone in the JCOG 9204 study. They found that the addition of adjuvant CT improved the disease-free survival rate (from 45 to 55%; *P* = 0.037) and the pN1 subgroup 5-year OS (52% vs. 38%, *P* = 0.041); however, there was no significant difference in the 5-year OS of the entire cohort (61% with CT vs. 52% with observation; *P* = 0.13) [25]. From these data, JCOG 9907 implied that neoadjuvant CT was more beneficial than surgery alone. Up to now, however, none of the randomized trials that compared neoadjuvant CT with surgery alone in esophageal SCC has demonstrated an increase in OS in those patients treated with neoadjuvant CT.

In an updated meta-analysis by Sjoquist and colleagues of nine randomized trials comparing neoadjuvant CT with surgery alone, in all histological subtypes an HR of 0.87 was reported (95% CI: 0.79 to 0.96; *P* = 0.05) [26]. There was no significant benefit for patients with SCC (HR: 0.92, 95% CI: 0.81 to 1.04; *P* = 0.18).

#### Neoadjuvant chemoradiotherapy

CT combined with RT was initially evaluated as a definitive treatment for patients identified as unable to proceed with surgery [27]. In combination, CT not only complements but increases the effect of RT in a process known as radiation sensitization, as well as complementing synergistic DNA damage, cell cycle synchronization, and inhibition of repair pathways [28]. In addition to increasing the efficacy of RT and thus controlling local tumor growth, CT theoretically also offers the ability to eradicate micrometastatic disease and decrease the risk of distant recurrence [29].

Several trials have investigated neoadjuvant concurrent CRT in esophageal SCC compared with surgery alone [30-36] (Table 3). The results of a phase III randomized controlled study (FFCD 9901) showed that neoadjuvant CRT with cisplatin and 5-FU did not improve OS but enhanced the postoperative mortality rate for patients with localized stage I or stage II esophageal cancer compared with surgery alone [31]. Another two studies reported no survival benefit with the addition of CRT with cisplatin and 5-FU regimens [32,33]. A larger study that randomized 256 patients with either AC or SCC to receive concurrent CRT or surgery alone reported no difference in OS between the two arms. However, a subgroup analysis demonstrated a significant improvement in progression-free survival (HR: 0.47, 95% CI: 0.25 to 0.86), although not in OS for the smaller subgroup of patients with SCC [34]. The Cancer and Leukemia Group B 9781 trial of neoadjuvant cisplatin and 5-FU in weeks 1 and 5 with RT (50.4 Gy in 28 fractions) compared with surgery alone closed to accrual prematurely having recruited only 56 of the planned 475 patients with resectable SCC or AC, owing to slow recruitment. Despite the limited sample size, 5-year OS was significantly improved from 16 to 39% in the trimodality arm and the pathological complete response rate was 40% [35]. Results from a recent multicenter phase III randomized trial (CROSS study) showed that neoadjuvant CRT with carboplatin and paclitaxel improved OS compared with surgery alone in patients with resectable (T2-3, N0-1, M0) esophageal or gastroesophageal junction cancers. The reported rate of R0 resection was higher in the CRT arm compared with the surgery alone arm (92% vs. 65%). The OS was significantly better for patients treated with CRT. Median survival was 49 months in the CRT arm compared with 26 months in the surgery alone arm. The 1-year, 2-year and 3-year survival rates were 82%, 67% and 59% respectively in the CRT arm and 70%, 52% and 48% respectively in the surgery-alone arm [36].

**Table 3:** Randomized controlled trials of neoadjuvant chemoradiotherapy versus surgery alone for esophageal squamous cell carcinoma

**Study**	**Histology**	**SCC (%)**	**Treatment**	***n***	**MS (months)**	**5-year OS (%)**	***P *value**
Bosset and colleagues [30]	SCC	100	C + 37 Gy	143	19	7	NS
			Surgery	139	19	9	
Lee and colleagues [32]	SCC	100	CF + 45 Gy	51	28	49^a^	NS
			Surgery	50	27	41^a^	
Burmeister and colleagues [34]	AC/SCC	35	CF + 35 Gy	128	22	17	NS
			Surgery	128	19	13	
Natsugoe and colleagues [33]	SCC	100	CF + 40 Gy	22		57	0.58
			Surgery	23		41	
Tepper and colleagues [35]	AC/SCC	25	CF + 50.4 Gy	30	54	39	<0.01
			Surgery	26	21	16	
Mariette and colleagues [31]	AC/SCC	66	CF + 45 Gy	97	32	49^a^	0.68
			Surgery	98	44	55^a^	
Gaast and colleagues [36]	AC/SCC	24	PC + 41.4 Gy	175	49	59^a^	0.011
			Surgery	188	26	48^a^	

^a^Three-year overall survival (OS). *AC* adenocarcinoma, *C* cisplatin, *CF* cisplatin + fluorouracil, *MS* median survival, *NS* not significant, *PC* paclitaxel + carboplatin, *SCC* squamous cell carcinoma.

The updated meta-analysis of 12 randomized compared neoadjuvant CRT with surgery alone. The HR for all-cause mortality for neoadjuvant CRT was 0.78 (95% CI: 0.70 to 0.88; *P* < 0.0001), the HR for SCC only was 0.80 (95% CI: 0.68 to 0.93; *P* = 0.004) and the HR for AC only was 0.75 (95% CI: 0.59 to 0.95; *P* = 0.02) [26]. Although this meta-analysis favors the neoadjuvant CRT approach for patients with SCC, this approach is also associated with significant treatment-related mortality and morbidity in this often malnourished population, who frequently have multiple smoking-related co-morbidities. The effectiveness of CRT with high pathological complete response rates prompted investigation of radical CRT as a definitive treatment modality to avoid surgical morbidity and mortality. The meta-analysis performed by Kranzfelder and colleagues, however, demonstrated that none of the randomized controlled trials was reporting an outcome of higher survival benefit after definitive CRT than neoadjuvant treatment followed by surgery, but treatment-related mortality rates were lower (HR 7.60; *P* = 0.007) than with neoadjuvant treatment followed by surgery or surgery alone [37]. On the other hand, Akutsu and colleagues investigated the clinical and pathologic results of neoadjuvant CRT in 78 patients that underwent an esophagectomy after neoadjuvant CRT. They found that the clinical evaluation for CRT does not reflect the pathologic effectiveness; even if clinical complete response was achieved, viable cancer cells were still present at the primary site in the majority of the population [38].

### Adjuvant treatments

#### Adjuvant radiotherapy

Adjuvant RT has also been evaluated in five small studies [39-43] (Table 4). A meta-analysis of these trials demonstrated no significant improvement in 5-year OS with the addition of RT to complete surgical resection [44].

**Table 4:** Randomized controlled trials of adjuvant radiotherapy versus surgery alone for esophageal squamous cell carcinoma

**Studies**	**Histology**	**SCC (%)**	**Treatment**	***n***	**MS (months)**	**5-year OS (%)**	***P *value**
Kunath and Fischer [39]	SCC	100	ART 50 to 55 Gy	23	9		NS
			Surgery	21	6		
Ténière and colleagues [40]	SCC	100	ART 45 to 55 Gy	102	18	19	NS
			Surgery	119	18	19	
Fok and colleagues [41]	SCC	100	ART 43 to 53 Gy	42	11	10	NS
			Surgery	39	22	16	
Zieren and colleagues [42]	SCC	100	ART 56 Gy	33		23^a^	NS
			Surgery	35		22^a^	
Xiao and colleagues [43]	SCC	100	ART 50 to 60 Gy	220		41	NS
			Surgery	275		32	

^a^Three-year overall survival (OS). *ART* adjuvant radiotherapy, *MS* median survival, *NS*, not significant, *SCC* squamous cell carcinoma.

Ténière and colleagues evaluated 221 patients with SCC of the middle to lower third of the esophagus who were randomized to adjuvant RT at a dose of 45 to 55 Gy versus observation [40]. They found that although local control improved from 15 to 30%, there was no survival benefit with the addition of adjuvant RT. Fok and colleagues also randomized 130 patients with esophageal carcinoma (SCC or AC) to observation versus adjuvant RT at a dose of 49.5 Gy in 3.5 Gy fractions [41]. They found that although local failure was reduced from 31 to 15% (*P* = 0.06), the median OS was actually worse in the adjuvant RT arm (8.7 months vs. 15.2 months, *P* = 0.02). This trial has been criticized, however, for the high dose per fraction that may have led to increased mortality in the radiation-containing arm. Zieren and colleagues randomized 68 patients with SCC to either adjuvant RT or surgery alone and found that adjuvant RT significantly increased the fibrotic stricture rate and did not improve OS or disease-free survival [42]. Malthaner and colleagues [44] performed a meta-analysis of 995 patients from five randomized trials of adjuvant RT versus observation. They found that there was no OS benefit with the addition of adjuvant RT, with a risk ratio for death at 1 year of 1.23 (95% CI: 0.95 to 1.59, *P* = 0.11). Suggestions therefore indicate there are few data to suggest that adjuvant RT offers any survival benefit. However, both Ténière and colleagues [40] and Zieren and colleagues [42] included patients with positive celiac nodes (stage M1a). These patients represent a cohort at much higher risk for distant failure and therefore are less likely to benefit from adjuvant RT. Finally, the meta-analysis included the above flawed trials.

Xiao and colleagues [43] randomized 495 patients with esophageal SCC to radical resection alone versus adjuvant RT at a total dose of 50 to 60 Gy in 2 Gy fractions. Once again, there was no survival benefit for the entire cohort with the addition of adjuvant RT, with a 5-year OS of 31.7% for surgery alone versus 41.3% for adjuvant RT (*P* = 0.447). When stratifying based on stage, however, there was a significant survival benefit with adjuvant RT for stage III patients, with an improvement in 5-year OS from 13.1 to 35.1%, (*P* = 0.003) but not for stage II patients. Similar to the findings mentioned above, Chen and colleagues retrospectively evaluated patients with thoracic esophageal SCC [45]. They reported that adjuvant RT was associated with a statistically significant improvement in survival, but only in those patients with three or more involved lymph nodes; no such association was found in patients with one or two involved lymph nodes.

In summary, the majority of the available evidence currently reveals that only selected locally advanced esophageal SCC are likely to benefit from adjuvant RT.

#### Adjuvant chemotherapy

Adjuvant CT with cisplatin-based regimens compared with surgery alone has been examined in three separate phase III trials [25,46,47] (Table 5). None of these trials reported a statistically significant difference in OS, although Ando and colleagues reported a 5-year OS advantage (52% vs. 38%, *P* = 0.041) in the pN1 subgroup patients [25].

**Table 5:** Randomized controlled trials of adjuvant chemotherapy versus surgery alone for esophageal squamous cell carcinoma

**Study**	**Histology**	**SCC (%)**	**Treatment**	***n***	**MS (months)**	**5-year OS (%)**	***P *value**
Pouliquen and colleagues [46]	SCC	100	CF	52	13		NS
			Surgery	68	14		
Ando and colleagues [47]	SCC	100	CV	100		45	NS
			Surgery	105		48	
Ando and colleagues [25]	SCC	100	CF	120		61	NS
			Surgery	122		52	

*CF* cisplatin + fluorouracil, *CV* cisplatin + vindesine, *MS* median survival, *NS* not significant, *OS* overall survival, *SCC* squamous cell carcinoma.

Two JCOG trials studied adjuvant CT for resected SCC of the esophagus. In the first study, 205 patients with resected SCC of the esophagus were randomized to receive two cycles of adjuvant CT with cisplatin 70 mg/m^2^ (day 1) and vindesine 3 mg/m^2^ (day 1) or observation only. No significant benefit in survival was detected from the addition of this adjuvant CT regimen (5-year OS: 48.1% vs. 44.9% with observation; *P* = 0.55) [47]. The follow-up JCOG 9402 study randomized 242 patients with resected SCC of the esophagus to two cycles of cisplatin 80 mg/m^2^ (day 1) and 5-FU 800 mg/m^2^ /day (days 1 to 5) or observation. The addition of this adjuvant CT regimen improved the disease-free survival rate (from 45 to 55%; *P* = 0.037) and the pN1 subgroup 5-year OS (52% vs. 38%, *P* = 0.041); however, there was no significant difference in the 5-year OS (61% with CT vs. 52% with observation; *P* = 0.13) [25].

#### Adjuvant chemoradiotherapy

The efficacy of adjuvant CRT has not been compared with surgery alone in a randomized trial in patients with esophageal SSC. The National Comprehensive Cancer Network does recommend postoperative adjuvant CRT for stages II and III esophageal AC. This recommendation is based on a randomized phase III trial by Macdonald and colleagues that found adjuvant CRT improved 3-year OS from 41 to 50% in AC of the stomach and gastroesophageal junction [48]. A small randomized trial of 45 patients compared adjuvant CT using cisplatin 50 mg/m^2^ (days 1 and 15) and 5-FU 300 mg/m^2^ /day (for 5 weeks) with adjuvant CRT after R0 resection of esophageal SCC. No significant difference in 5-year OS was demonstrated (38% CT vs. 50% CRT; *P* = 0.97), but the study was limited by the small sample size [49].

## Discussion

None of the randomized trials that compared neoadjuvant RT or CT with surgery alone in esophageal SCC has demonstrated an increase in OS in those patients treated with neoadjuvant RT or CT. Neoadjuvant CRT has been accepted recently for esophageal cancer because such a regimen increases the rates of R0 resection and local tumor control. The updated meta-analysis also favors the neoadjuvant CRT approach for patients with SCC [26]. Another recent meta-analysis demonstrated that the esophageal SCC in Europe and in the USA benefited from neoadjuvant CRT; however, patients in Asia did not benefit [50]. Ethnic difference makes it necessary to re-evaluate the role of neoadjuvant CRT in patients with SCC in Asia.

Phase III trials have compared adjuvant RT with surgery alone without demonstrating any OS benefit, except in stage III patients (35.1% vs. 13.1%, *P* = 0.003) [44]. The phase II nonrandomized trial for patients with node-positive (T3 or T4) tumors indicated that the outcomes of adjuvant CRT are better than the historical outcomes with surgery alone [49]. The retrospective study indicated that adjuvant RT was associated with a statistically significant improvement in survival, but only in those patients with three or more involved lymph nodes [45]. The addition of adjuvant CT only improved the pN1 subgroup 5-year OS [25]. The majority of the available evidence therefore currently reveals that only selected locally advanced esophageal SCC are more likely to benefit from the adjuvant therapy. The National Comprehensive Cancer Network recommends upfront surgery only for select patients with clinical T1 disease. For all other disease stages, CRT, preoperative CRT, or preoperative CT is recommended [51]. The current evidence reveals that primary surgery followed by adjuvant therapy should be used cautiously in clinical practice until appropriately powered randomized trials confirm the adjuvant treatment results of this disease.

Although both neoadjuvant CRT and adjuvant CRT have emerged as more useful options for the treatment of resectable esophageal SCC, it is hard to identify which is better based on current knowledge in the lack of head-to-head randomized controlled trials. Designing proper multidisciplinary studies that include use of novel systemic chemotherapies, optimizing radiation techniques, and identifying patient and tumor-specific markers predictive for response and/or toxicity remains an urgent need for the future. The approach is currently being explored in China by investigators of the ZTOG1201 trial, a multicenter phase II trial of neoadjuvant and adjuvant CRT in locally advanced esophageal cancer (NCT01463501).

Future trials should investigate further ways to improve upon current loco-regional control and survival. Opportunities for development include use of novel chemotherapies. Docetaxel was recently reported as a powerful anticancer agent for esophageal cancer in numerous trials. Cisplatin plus docetaxel, with or without 5-FU, has demonstrated activity in patients with locally advanced EGI or metastatic esophageal SCC [52-54]. A small-sample retrospective study has also showed that preoperative combined CT with docetaxel plus cisplatin and 5-FU followed by surgery provided a high response rate and a favorable survival benefit in patients with resectable esophageal SCC [55]. In the docetaxel plus cisplatin and 5-FU group, the overall response rate was 92.3%; 30.8% patients had complete response, 61.5% partial response, 7.7% stable disease, and 0 progressive disease. To validate the clinical significance of this novel CT regimen, large-sample randomized trials are essential.

## Conclusion

None of the randomized trials that compared neoadjuvant RT or CT with surgery alone in esophageal SCC has demonstrated an increase in OS in those patients treated with neoadjuvant RT or CT. Neoadjuvant CRT has been accepted recently for esophageal cancer because such a regimen offers great opportunity for margin negative resection, improved loco-regional control and increased survival, and should be an optional treatment paradigm. The majority of the available evidence currently reveals that only selected locally advanced esophageal SCC are more likely to benefit from the adjuvant therapy. The focus of future trials should be on identification of the optimum regimen and should aim to minimize treatment toxicities and effect on quality of life, as well as attempt to identify and select those patients most likely to benefit from specific treatment options.

## Abbreviations

AC: adenocarcinoma; CI: confidence interval; CT: chemotherapy; CRT: chemoradiotherapy; 5-FU: fluorouracil; HR: hazard ratio; JCOG: Japan Clinical Oncology Group; OS: overall survival; RT: radiotherapy; SCC: squamous cell carcinoma.

## Competing interests

The authors declare that they have no competing interests.

## Authors’ contributions

All authors contributed equally to this work and approved the final manuscript.
